# The role of HIF-1α-VEGF pathway in bronchiolitis obliterans after lung transplantation

**DOI:** 10.1186/s13019-019-0832-z

**Published:** 2019-01-29

**Authors:** Haichao Xu, Abudumailamu Abuduwufuer, Wang Lv, Zhenyu Zhou, Yunhai Yang, Chong Zhang, Jian Hu

**Affiliations:** Thoracic Surgery, The First Affiliated Hospital, Zhejiang University, 79 Qingchun Road, Hangzhou, China

**Keywords:** Lung transplantation, Bronchiolitis obliterans, Fibrosis, Hypoxia-inducible factor 1, alpha subunit, Vascular endothelial growth factor a

## Abstract

**Background:**

Graft function may be affected if the organ is exposed to hypoxia. We hypothesized that bronchiolitis obliterans (BO) after lung transplantation is associated with hypoxia-inducible factor-1α (HIF-1α). This study compares the expression of HIF-1α and its downstream proteins in allograft and isograft to explore the relationship between this pathway and BO in rats.

**Material and methods:**

We performed an orthotopic left pulmonary transplant model using the tri-cuff vascular anastomosis method and evaluated the histopathology, including the severity of fibrosis (SF). The expression of HIF-1α, VEGF-A, and VEGFR-2 was accessed by immunohistochemistry.

**Results:**

The imageology and pathology showed that the allogenic model developed BO 90 days after the operation. The percentages of a high expression of HIF-1α, VEGF-A, and VEGFR-2 in the allogeneic group were 77.27, 63.64, and 68.18% higher than in the isogeneic group, respectively. The SF score was highest in the allograft and was positively correlated with the expression of the proteins.

**Conclusion:**

This model can simulate human BO after lung transplantation. The expression of HIF-1α and its downstream proteins in post-transplantation was up-regulated, suggesting that activation of the HIF-1α-VEGF pathway might be involved in the occurrence and prognosis of BO.

## Background

At present, lung transplantation is still the best treatment for end-stage lung disease. Chronic rejection, the pathological manifestation of which appears as bronchiolitis obliterans (BO), also known as bronchiolitis obliterans syndrome (BOS), is the main factor affecting the long-term survival of patients who have undergone lung transplantation [1, 2]. Based on “The Registry of the International Society for Heart and Lung Transplantation: Thirty-second Official Adult Lung and Heart-Lung Transplantation Report,” it was estimated that 5 years after lung transplantation 50% of the recipients would have developed BOS and 76% would have developed BOS 10 years after transplantation [3]. In recent years, the study of lung transplantation and BOS has been facilitated by the establishment of several relatively mature experimental models of orthotopic left-lung transplantation in rats and mice, based on the cuff technique or other anastomotic methods [4–6].

Hypoxia-inducible factor-1 (HIF-1) is a transcription factor that becomes activated under hypoxia. It is involved in the immune/inflammatory reaction in various pathophysiological statuses, encoding or regulating more than 50 kinds of proteins including erythropoietin, glycolytic enzymes, induced-NOS, and vascular endothelial growth factor (VEGF) [7–10]. HIF-1, as a heterologous dimer, is composed of a functional alpha subunit and a structural beta subunit, and its biological effect is achieved by HIF-1 alpha (HIF-1α), which mediates the recruitment of bone marrow-derived angiogenic cells to increase arterial remodeling and angiogenesis during transplantation. Among the numerous target proteins associated with HIF-1, the protein of most concern is the VEGF, which is well-known for its pro-angiogenic and pro-fibrogenic effects. It has six isoforms, from VEGF-A to VEGF-F, where VEGF-A specifically promotes the division and proliferation of vascular endothelial cells and the migration of inflammatory cells. It is confirmed that HIF-1α signaling inhibition can downregulate VEGF induced fibrinogenesis and angiogenesis in vitro [11]. Recent studies have found that VEGF-A can bind to VEGF receptor-2 (VEGFR-2) to enhance vascular permeability, promote the expression of cytokines, and induce the inflammatory cells’ chemotaxis, which may play a role in the inflammatory response of transplanted organs [8, 12, 13].

The oxygen concentration in the graft is different from normal after transplantation due to the change in anatomic structures and the involvement of immune, inflammation, and other internal environmental factors [7, 14–16]. At present, it is not clear whether this change would affect the expression of HIF-1 and its downstream proteins, and relate to the rejection of the allograft. Thus, we studied the expression of HIF-1α and VEGF-A, and the relationship between these proteins and BO, in rats after orthotopic left-lung transplantation.

## Material and methods

### Animals

Fischer 344 (F344) and Lewis (LEW) male rats were purchased from the experimental animal center at Zhejiang Academy of Medical Sciences. They were reared in the SPF laboratory of the Key Laboratory of Multiorgan Transplantation at the Chinese Ministry of Health (The First Affiliated Hospital, Zhejiang University, Hangzhou). Animals weighing 250–300 g were used as both donors and recipients. All experimental animal protocols were reviewed and approved by the ethics committees at The First Affiliated Hospital and the Experimental Animal Center, Zhejiang University, China.

### Orthotopic lung transplantation

The orthotopic left-lung transplantation was performed in the F344-to-Lewis rat strain combination as the allogeneic group (*n* = 24). As described in previous studies [4], the F344 rats (RT1^1v1^) were used as donors and the Lewis rats (RT1^1^) as recipients. In the control group (*n* = 12), both the donors and the recipients were the Lewis rats. We had two trained laboratory technicians who dealt with donors and recipients, respectively. To reduce the time of cold ischemia and anesthesia of the recipient as much as possible, when one technician separates the donor lung hilar, the other one can start recipient surgery, so that pulmonary hilar anastomosis can be performed as soon as possible. Recipient operation time is from cutting the recipient rat skin, to the end of the skin suture. Warm ischemia time is composed of two parts, the first part is from donor rats superior vena cava disconnection to lung lavage completion, the second part starts with anastomosis, ends of the recipient pulmonary artery opening. Cold ischemia time is the transition time between the first and the second part of the warm ischemia time. Recipients of all groups received the same drug treatment, and specifically, with ciclosporin (Sandimmune, 50 mg/ml; Novartis, Nürnberg, Germany) on the first 10 days and lipopolysaccharide (LPS; L2654, from *Escherichia coli*, 026:B6, Sigma-Aldrich, Steinheim, Germany) once on the 28th day after transplantation.

### Histology

The recipients were sacrificed 90 days after transplantation. The lungs were immediately fixed in situ by an intratracheal instillation of 4% formaldehyde for 24 h with subsequent paraffin embedding. The tissue sections were prepared and stained with hematoxylin-eosin (HE) and Masson’s trichrome staining. The staging of the lung allograft rejection was completed according to the International Society for Heart and Lung Transplantation guidelines [2]. The severity of fibrosis (SF) was measured on an arbitrary scale of 0 to 4 for reference [17, 18].We conducted the histopathologic analysis in a blinded fashion.

### Immunohistochemistry

Immunohistochemistry staining was performed by the streptavidin peroxidase complex method to detect HIF-1α, VEGF-A, and VEGFR-2. The immunohistochemistry score was the product of the staining intensity and percentage of positive cells. The staining intensity was graded from 0 to 3+ (0 for colorless, 1+ for pale yellow, 2+ for brown-yellow, and 3+ for saddle-brown). The score for the percentage of positive cells was from 0 to 4+ (0 for negative, 1+ for fewer than 10% positive cells, 2+ for 10–50% positive cells, 3+ for 51–75% positive cells, and 4+ for over 75% positive cells). The immunohistochemistry score ≥ 3 was considered positive immune response.

### Statistical analysis

All numerical data of the experimental results were presented as mean ± standard deviation and categorical data as count and percentage. The statistical analyses were accomplished using the GraphPad Prism 5.0 (GraphPad Software, San Diego, CA). Tests were performed with the Pearson’s chi-square test for count data and the student T test for measurement data. All *p*-values were two-tailed and differences were considered statistically significant at *p* < 0.05.

## Results

### Animal model of lung transplantation

We have successfully completed a total of 35 rat lung transplantations, with an average operation time for the recipient of about 27 min and a 97.2% success rate (35/36). Cold ischemia time is about 10 min, and warm ischemia time is about 12.5 min. There were two deaths in the allogeneic group: one was caused by a pleural hemorrhage on the third day after surgery and the other by a digestive obstruction on the fiftieth day after the operation. In the isogeneic group, one rat’s pulmonary vein was torn during the operation, eventually leading to death by hemorrhagic shock and another died 3 days after the surgery due to a pulmonary embolism. So finally, we have 22 allogeneic recipients in the allogeneic group and 10 isogenic recipients in the control group.

**Fig. 1:**
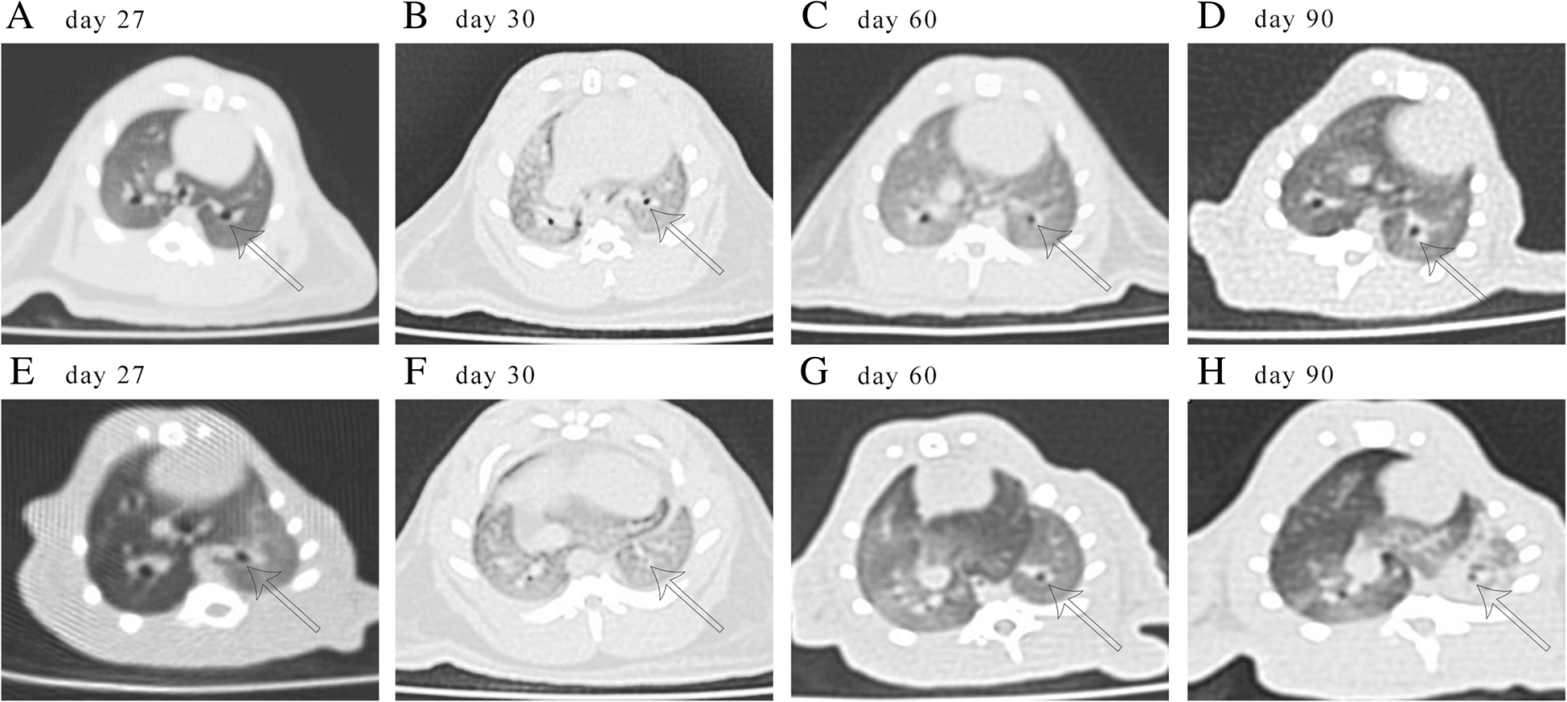
High-resolution computed tomography imaging of lung isografts (**a**-**d**) and allografts (**e**-**h**). The chest computed tomography imaging was performed in the anesthetized animal on 27 (**a** and **e**), 30 (**b** and **f**), 60 (**c** and **g**), and 90 (**d** and **h**) days after transplantation. The white arrows in left pulmonary point to the algate aerated bronchus

Each recipients received the chest computed tomography scan at specific points [19]. We selected a typical example from each group (Fig. 1). On day 27, the computed tomography images of the two groups were similar for clear lung fields and unobstructed bronchus (Fig. 1a and e). On day 30 after surgery, the diffuse infiltration of bilateral lung parenchyma was seen in every model and the surrounding of the bronchus was more serious as the LPS was applied intratracheally (Fig. 1b and f). Sixty days after transplantation, lung fields appeared clearer than the previous scan (Fig. 1c) while the allogenic left lungs still had an increased lung field density and emphysema appeared in the right native lungs (Fig. 1g). Ninety days postoperatively, the lung fields of the isografts and the right native lungs of the allogenic recipients were clear (Fig. 1d and h). However, the allogenic left lungs showed disseminated high density shadows with local atelectasis and mediastinum shifted to the left (Fig. 1h).

**Fig. 2:**
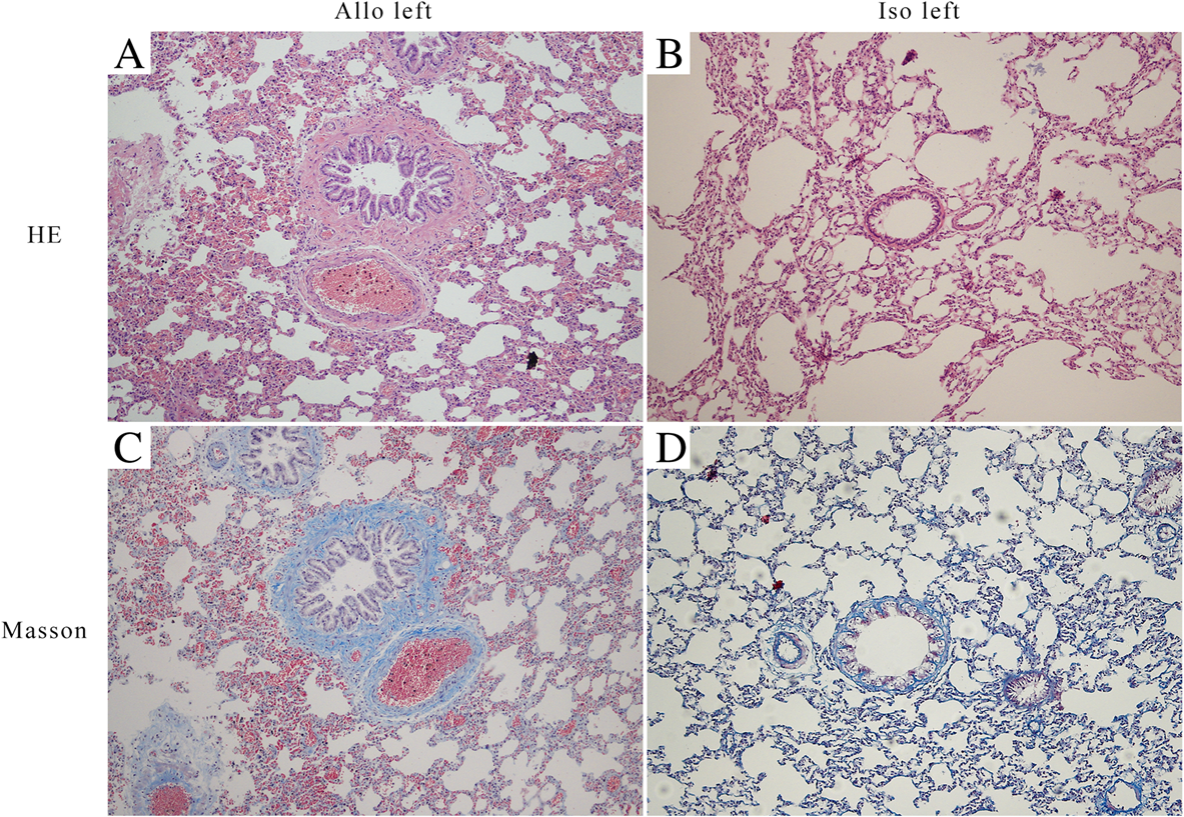
Histopathology of lung allografts (**a** and **c**) and isografts (**b** and **d**) in HE and Masson’s trichrome staining. The magnification of pictures was 200×. HE, hematoxylin-eosin staining; Allo left, allogenic left lung; Iso left, isogenic left lung

By observing the paraffin sections stained with HE under a light microscope, we found airway remodeling in sections of the allogenic left lungs, and the bronchial lumina displayed severe stenosis or occlusion (Fig. 2a), obviously different from the native right lungs and the isografts (Fig. 2b). The inflammatory cells predominantly consisted of mononuclear leukocytes that infiltrated the bronchial mucosa and the area surrounding the bronchus. Around the bronchioles, there are proliferative bronchial mucosal epithelial cells and smooth muscle cells. In addition to bronchiolitis, It may be accompanied by abnormal thickening of arterial intima (Fig. 2a). Fibrous connective tissue, such as collagen, was stained in blue when Masson’s trichrome staining was applied. We found dense bands of collagen surrounding bronchioles, replacing large areas of lung parenchyma, in sections of the allogenic left lungs (Fig. 2c), which differ from the isografts (Fig. 2d).

### Expression of HIF-1α increased in the transplanted lung

**Fig. 3:**
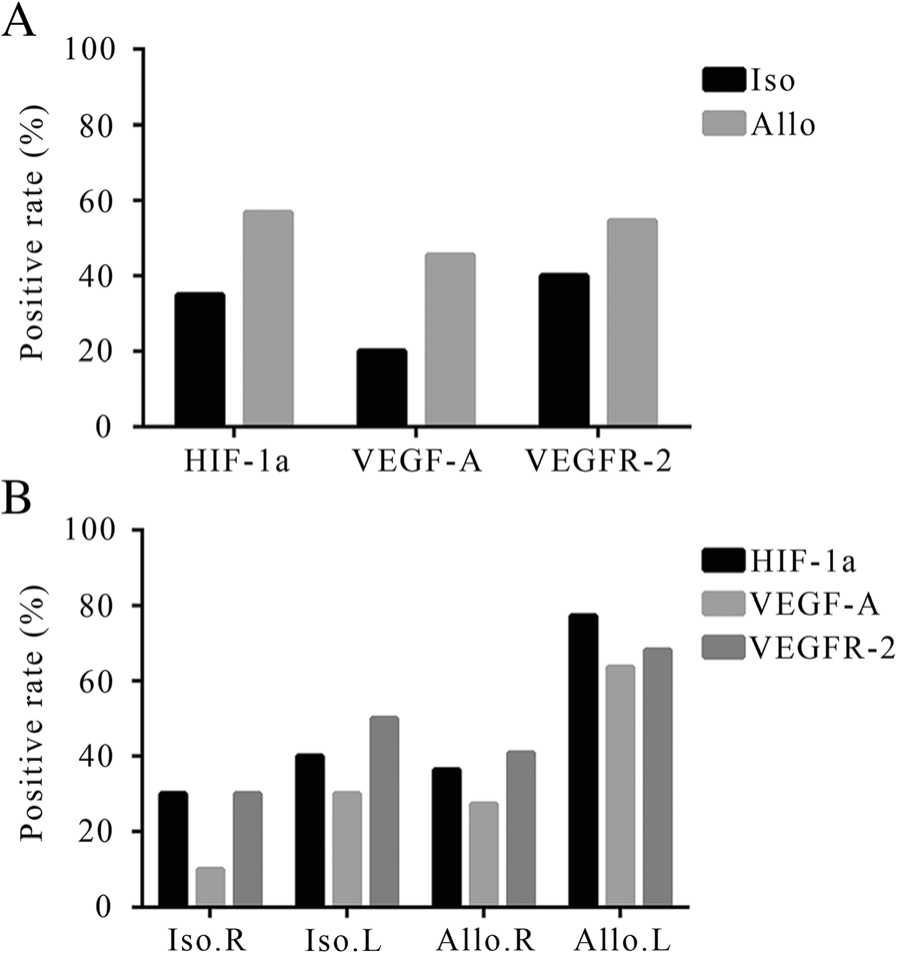
Expression of HIF-1α, VEGF-A and VEGFR-2. The positive rate of related protein’s expression is different between **a** the isografts (*n* = 10) and the allografts (*n* = 22), and **b** in different position of lung. Iso, isograft; Allo, allograft; HIF-1α, Hypoxia inducible factor-1α; VEGF-A, vascular endothelial growth factor-A; VEGFR-2, vascular endothelial growth factor receptor-2; R, right lung; L, left lung

**Fig. 4:**
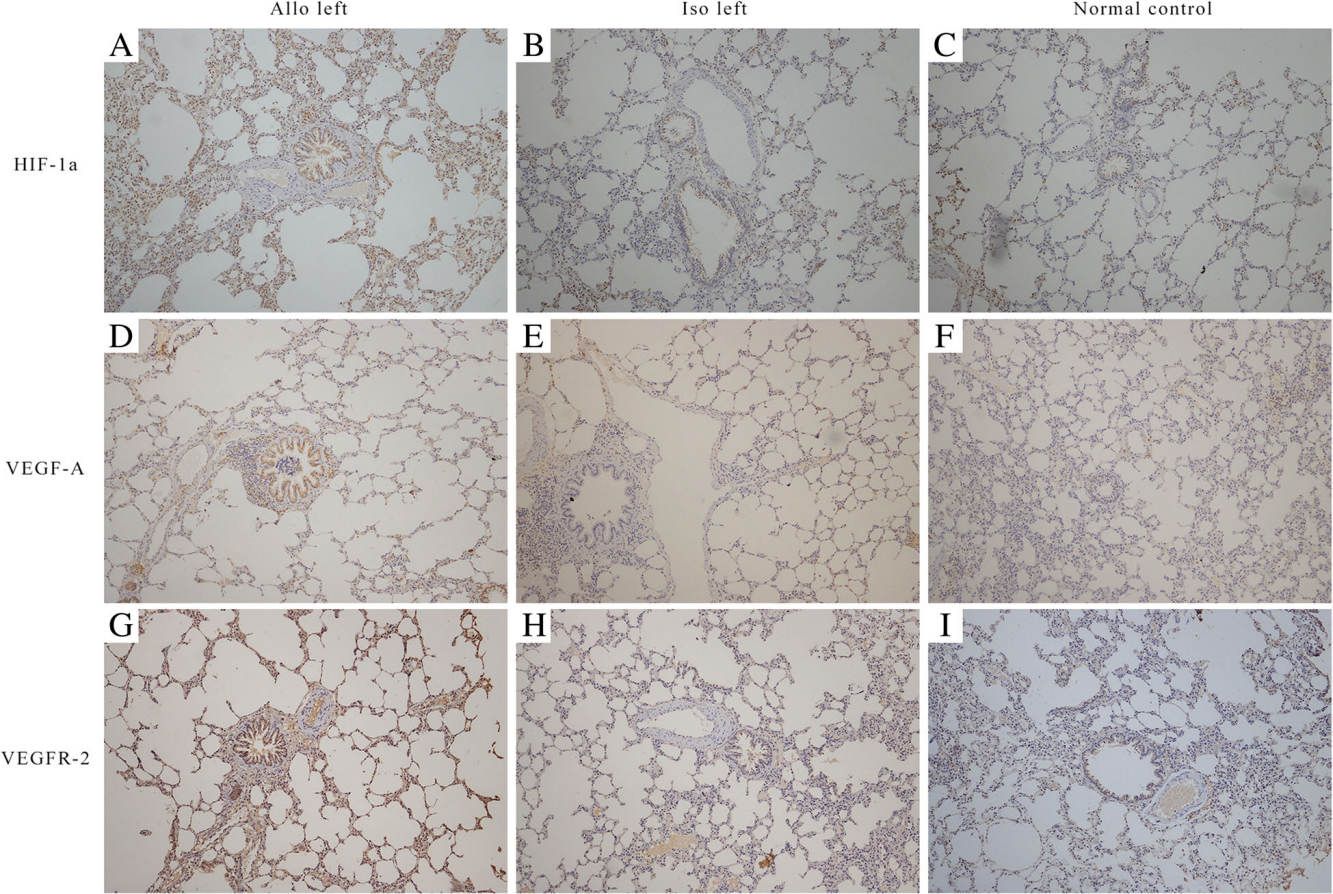
Immunohistochemistry of HIF-1α, VEGF-A and VEGFR-2 in lung grafts or in situ. Protein expression was assessed as positive (**a**, **d** and **g**) or negative (**b**, **c**, **e**, **f**, **h** and **i**) depending on the immunohistochemistry score in different position. Positive immune response group had more brown-staining cells in particular areas than the negative group. The magnification of pictures was 200×. HIF-1α, Hypoxia inducible factor-1α; VEGF-A, vascular endothelial growth factor-A; VEGFR-2, vascular endothelial growth factor receptor-2

We used immunohistochemical staining with antigen-specific antibodies to better quantify and locate the proteins. As described above, we divided the pathological sections into positive or negative immune response groups, also called high- or low-expression groups, according to the immunohistochemistry score. The percentages of high-expression of HIF-1α, VEGF-A, and VEGFR-2 in the allogeneic group were 56.82, 45.45, and 54.55%, compared with 35, 20, and 40% in the isogeneic group (Fig. 3a), respectively. When we counted the data on the left and right lungs individually, we found that the positive rates of high-expression of HIF-1α, VEGF-A, and VEGFR-2 in the isograft were similar to the right lung in situ of the two groups, but significantly lower than in the allograft (77.27, 63.64, and 68.18%, respectively) (Fig. 3b). Simultaneously, we found that the locations of the different proteins inside the cells were not exactly the same. HIF-1α was mainly expressed in the nucleus because it was a transcription factor, which mainly functions in the nucleus, whereas VEGF-A and VEGFR-2 were mainly observed in the cytoplasm (Fig. 4).

### Expression of HIF-1α related to the severity of pulmonary fibrosis

**Fig. 5:**
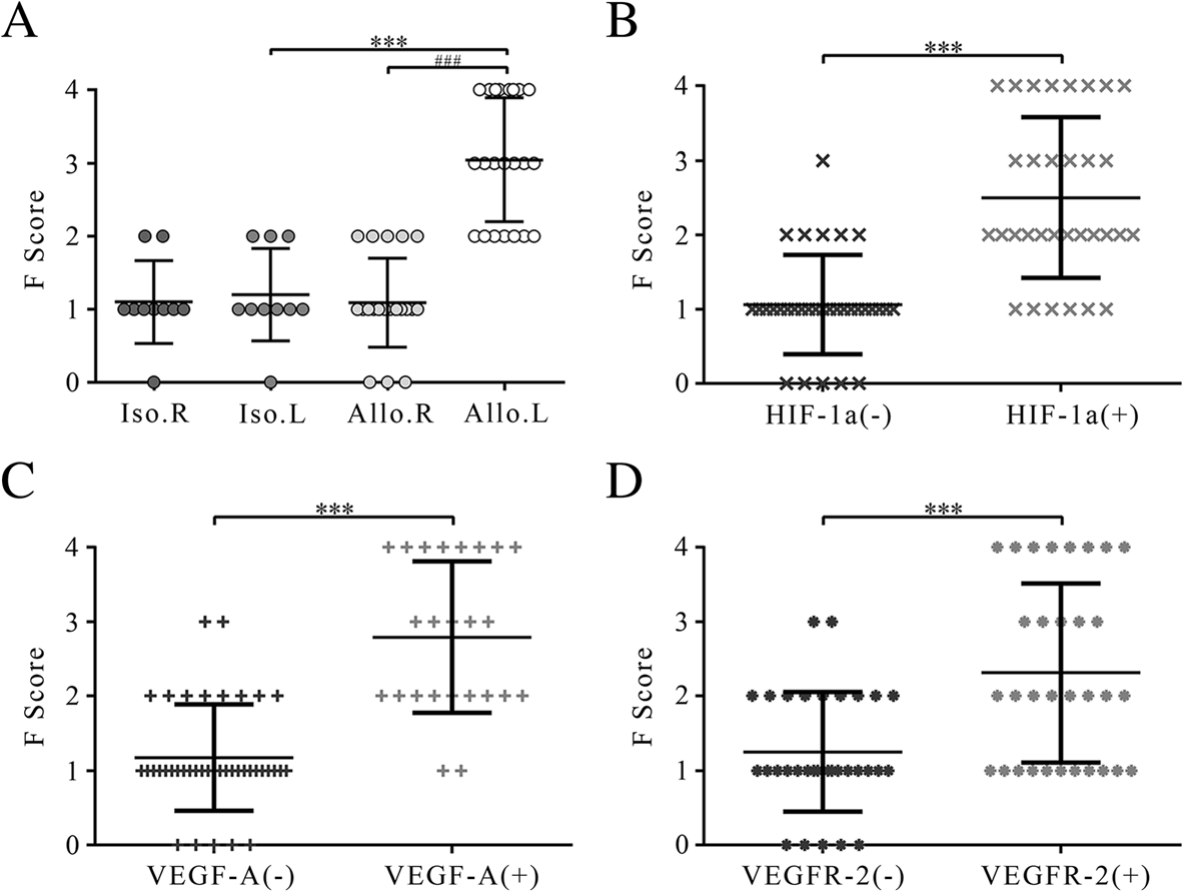
The severity of pulmonary fibrosis of allografts and isografts. Expression of HIF-1α, VEGF-A and VEGFR-2 is related to the severity of pulmonary fibrosis. **a** n = 10 (Iso) and n = 22 (Allo); **b** *n* = 32 (HIF-1α(−)) and *n* = 32 (HIF-1α(+)); **c** *n* = 40 (VEGF-A(−)) and *n* = 24 (VEGF-A(+)); **d** *n* = 32 (VEGFR-2(−)) and *n* = 32 (VEGFR-2(+)); All data shown are the mean ± standard deviation. ^***^p < 0.01 and ^###^*p* < 0.01 with T test. F score, The severity of fibrosis score; Iso, isograft; Allo, allograft; HIF-1α, Hypoxia inducible factor-1α; VEGF-A, vascular endothelial growth factor-A; VEGFR-2, vascular endothelial growth factor receptor-2; R, right lung; L, left lung

The severity of pulmonary fibrosis, evaluated in the Masson’s trichrome staining, was mainly dependent on the thickness and number of fibers in the connective tissue. The SF of the allograft was 3.05 ± 0.84, apparently higher than the right lung in situ (1.09 ± 0.61, *P* = 3.60 × 10^− 9^) and the left lung of the isograft (1.16 ± 0.62, *P* = 5.39 × 10^− 7^), respectively. And, there were no statistical difference between the left and right lung of the isograft (1.16 ± 0.62, 1.10 ± 0.57) (Fig. 5a).

Then, all samples were divided into two groups to compare whether there were statistical differences in SF, based on whether the immunohistochemistry score ≥ 3 of the corresponding protein. The results showed that the SF was higher in samples with high protein expression (HIF-1α: 2.50 ± 1.09 vs. 1.06 ± 0.67, *P* = 3.88 × 10^− 10^; VEGF-A: 2.79 ± 1.03 vs. 1.18 ± 0.71, *P* = 7.53 × 10^− 13^; VEGFR-2: 2.31 ± 1.19 vs. 1.25 ± 0.81, *P* = 6.13 × 10^− 10^) (Fig. 5b-d). These results indicated that the expression of HIF-1α, VEGF-A, and VEGFR-2, and the severity of pulmonary fibrosis, was positively correlated.

### Correlation between HIF-1α, VEGF-A, and VEGFR-2

By calculating the correlations among HIF-1α, VEGF-A, and VEGFR-2, we found that HIF-1α and VEGF-A, VEGF-A and VEGFR-2, and HIF-1α and VEGFR-2 were all positively correlated (Table 1).

**Table 1:** Correlation between HIF-1α, VEGF-A and VEGFR-2

Parameters	n	VEGF-A (−) (*n* = 40)	VEGF-A (+) (*n* = 24)	χ^2^	*p*-value
HIF-1α (−)	32	30 (20)	2 (12)	26.67	2.41 × 10^−7^
HIF-1α (+)	32	10 (20)	22 (12)		
Parameters	n	VEGFR-2 (−) (*n* = 32)	VEGFR-2 (+) (*n* = 32)	χ^2^	*p*-value
HIF-1α (−)	32	22 (16)	10 (16)	9.00	2.70 × 10^−3^
HIF-1α (+)	32	10 (16)	22 (16)		
Parameters	n	VEGFR-2 (−) (*n* = 32)	VEGFR-2 (+) (*n* = 32)	χ^2^	*p*-value
VEGF-A (−)	40	26 (20)	14 (20)	9.60	1.95 × 10^−3^
VEGF-A (+)	24	6 (12)	18 (12)		

All samples are from both side of all recipient lung (the number of allogeneic group is 44 and the number of control group is 20) and divided into two groups based on whether the immunohistochemistry score ≥ 3 of the corresponding protein. (−) represents immunohistochemistry score < 3; (+) represents immunohistochemistry score ≥ 3; *HIF-1α* Hypoxia inducible factor-1α, *VEGF-A* Vascular endothelial growth factor-A, *VEGFR-2* Vascular endothelial growth factor receptor-2

## Discussion

The aim of our study was to explore the relationship between BO after lung transplantation in rats and the expression of HIF-1, etc. In the pretest, we searched for the appropriate animal model. Based on the literature [6, 20], we tried several models and found that there were different outcomes in the allografts for different strains. For instance, when Sprague-Dawley rats were used as recipients, there were very few rejections, yet when Brown Norway rats acted as recipients, the grafts were prone to acute rejection. As for trachea transplantation, although it is similar to BOS in immunology and morphology, we thought that the orthotopic lung transplantation model can better simulate chronic rejection if technical difficulties can be overcome. Finally, we selected the F344-to-Lewis rat strain combination as reported by Atanasova et al. [4] Based on our results, we believe that the chronic rejection outcomes demonstrated by this model resemble the 2007 revised criteria for the diagnosis of lung rejection [2]. On the ninetieth day after transplantation, the allograft sections showed bronchial mucosal epithelial hyperplasia with a large number of inflammatory cells infiltrating the bronchial mucosa and the area surrounding the bronchus. This was accompanied by the presence of coarse collagen and a large amount of proliferative smooth muscle. In addition to the proliferation of arterial intima, this resulted in concentric or eccentric stenosis or occlusion of the bronchial lumina. This morphology is similar to the BOS of human lung transplantation.

Chronic rejection of lung transplantation is the main limitation for long term survival of lung transplantation patients, which is associated with airway fibrosis. Studies demonstrated that the airways might be relatively hypoxic after lung transplantation, which had an influence upon the metabolism of graft [7, 16]. In our study, we found that the expression of HIF-1α, VEGF-A, and VEGFR-2 in the allografts was more than isografts. As is well-known, the HIF-1α was an important nuclear transcription factor that can be activated under hypoxic conditions to mediate increased transcription of genes involved in angiogenesis, growth factor signaling and oxygen transport to either increase O_2_ delivery or decrease O_2_ consumption [7, 10, 21, 22]. HIF-1–mediated transcriptional responses activate the expression of angiogenic cytokines and growth factors that stimulate angiogenesis by recruiting bone marrow-derived angiogenic cells as well as endothelial cells. The recent studies have shown that HIF-1α has a crucial role in critical aspects of cell metabolism and mammalian embryogenesis, and contributes to pathogenesis for a variety of diseases, such as hereditary erythrocytosis, pulmonary arterial hypertension, cancer and airway constrictive diseases [23].

In the acute phase of hypoxia, the tissue’s blood supply could mainly be affected by the angiotensin system and, when entering the chronic hypoxia process, this could compensate for a lack of oxygen through vascular remodeling. VEGF-A and VEGFR2 are a pair of ligands that induce angiogenesis in vivo. Abraham, Paulus, and others have found that early in transplantation, even in the donor organs preservation period, the level of HIF-1α in grafts was raised, and the transcription of the VEGF-A gene was up-regulated. This would directly affect the vascular permeability and the degree of edema after transplantation and, ultimately, the outcomes of the grafts [9, 12, 13, 24]. Our team expects that although HIF-1α, VEGF-A, and other proteins were up in the early stage of transplantation, their influence was perhaps not on the allograft rejection, but on increasing the vasopermeability and the recruitment of inflammatory cells. Due to bronchial arteries are not re-established, which are the main arterial blood supply for pulmonary, the grafts would be in hypoxemia after transplant surgery [13, 16, 22]. As a result, we suppose that if the state of hypoxia had not been changed, HIF-1α and downstream proteins might have had a continuous high expression. However, as there was no access to any relevant literature, our team completed the experiment and the results confirmed our hypothesis.

In other solid organ transplantation studies, researchers have found that HIF-1α, VEGF, and NOS were released in the anoxic condition. Macrophages, monocytes, and other inflammatory cells were then recruited and induced to secrete cytokines, such as transforming growth factor-1β, thrombospondin-1 and so on, which might promote the occurrence of graft fibrosis [7, 11, 13, 14, 24–27]. Researchers have recently used normal human bronchial epithelial cells in vitro to confirm that HIF-1a is associated with pulmonary fibrosis after transplantation [11]. Yet, Jiang et al. have shown a pro-angiogenic role of HIF-1α to mediates airway microvascular repair and thereby attenuate airway fibrotic remodeling by using a model of orthotopic tracheal transplantation. Studies demonstrated that chronic anoxia impaired HIF-1-dependent VEGF expression in graft [10]. When exogenous intervention upregulated HIF-1α, graft airway perfusion accelerated recovery by increasing expression of angiogenic factors and recruitment of host bone marrow-derived angiogenic cells to the airway [28]. The contradiction between the complex mechanisms and the phenomenons may be due to the different periods of research objects, as well as the characteristics of self-protection and the limitations of self-repair. In this way, it would be of great significance to explore the role of HIF-1α in different models and stages, for its apparently opposing roles.

According to the revision of the 1996 working formulation for the standardization of nomenclature in the diagnosis of lung rejection [2], BO is defined as a progressive fibrous obliteration of the small airways, an all-or-none diagnosis, with no index for judging the degree of severity. Thus, to more comprehensively assess the extent of graft rejection, we introduced the arbitrary scale of severity of fibrosis to score the sections [17, 18]. We found that the expression of HIF-1α, VEGF-A, and VEGFR2 was related to the degree of graft fibrosis. We confirmed a link between the HIF-1α-VEGF-A pathway and BO, in a long-term survival model of orthotopic lung transplantation in rats for the first time.

## Conclusions

We focused on the status of HIF-1α, VEGF-A, and VEGFR2 in BO after lung transplantation and found a link between these three proteins. According to our data, we think that VEGF-A, as a target protein of HIF-1, has specific binding to the VEGFR2 transmembrane protein, activating downstream secondary messengers to regulate the transcription of the relative genes involved in angiogenesis, pro-fibrogenesis, and other pathophysiological manifestations. In conclusion, in our model of orthotopic left-lung transplantation, BO was observed and the expression of HIF-1α, VEGF-A and VEGFR2 in the allograft tissue was up-regulated. It is thus suggested that the activation of the HIF-1α-VEGF pathway may be involved in the development and prognosis of BO after lung transplantation.

## Abbreviations

BO: Bronchiolitis obliterans
BOS: Bronchiolitis obliterans syndrome
F344: Fischer 344
HE: Hematoxylin-eosin
HIF-1α: Hypoxia inducible factor-1 alpha
LEW: Lewis
LPS: Lipopolysaccharide
Ltx: Lung transplantation
SF: Severity of fibrosis
VEGF-A: Vascular endothelial growth factor-A
VEGFR-2: Vascular endothelial growth factor receptor-2
